# Commentary: Heart rate variability and self-control–A meta-analysis

**DOI:** 10.3389/fpsyg.2016.00653

**Published:** 2016-05-04

**Authors:** Sylvain Laborde, Emma Mosley

**Affiliations:** ^1^Institute of Psychology, German Sport University CologneCologne, Germany; ^2^UFR STAPS, EA 4260, University of CaenCaen, France; ^3^Bournemouth UniversityBournemouth, UK

**Keywords:** vagal tone, parasympathetic nervous system, heart rate variability, self-regulation, parasympathetic activity

In relation to the paper “Heart rate variability and self-control: a meta-analysis,” we have recommendations that may be of importance when considering future research on HRV and self-control. The following commentary will cover three main points which consist of: (1) a systematic use of the terms “vagal/parasympathetic” instead of heart rate variability (HRV), as vagal tone is the real physiological process related to self-control (Vagal tone is a synonym of parasympathetic activity, given the vagus nerve is the main nerve of the parasympathetic nervous system); (2) a systematic investigation/acknowledgment of vagal tone at rest and its reactivity—further than resting HRV; (3) the strict selection of studies having self-control performance assessed concomitantly with vagal tone. Below we elaborate on these points, with specific recommendations for researchers.

1. The main point we would like to raise is the misleading focus of this meta-analysis. When considering self-control, the focus should not be about HRV—which encompasses the potential calculation of dozens of parameters in the time-domain, frequency-domain, as well as non-linear indices (Malik, 1996; Shaffer et al., 2014)—but about vagal tone, which is never fully acknowledged by the authors. Vagal tone is the real physiological mechanism that is linked with self-control, like acknowledged by both the neurovisceral integration model (Thayer et al., 2009) and the polyvagal theory (Porges, 2007). If the meta-analysis strictly followed the two theoretical backgrounds, it should have exclusively focused on HRV parameters reflecting vagal tone, for example the root mean square of the successive differences (RMSSD) and the high-frequencies (HF). SDNN, an additional HRV parameter mentioned in the meta-analysis, does not reflect vagal tone (Malik, 1996), and has therefore no theoretical reason to be linked to self-control, which is rather confusing for the reader. More critically, vagal tone is crucially missing from the keyword search. Neither “vagal,” “parasympathetic,” “RMSSD,” nor “HF” were included as keywords for the studies' searching process of this meta-analysis, which would omit studies relating to self-control when reported as vagal or parasympathetic tone and not having “heart rate variability” or the associated terms in the abstract or keywords. We would then recommend researchers investigating self-control to shift the focus from HRV to vagal tone, and include “vagal,” “parasympathetic,” “RMSSD,” and “HF” in their search rule.
2. The second point we would like to raise is the paper's focus on resting HRV and the exclusion of studies related to HRV reactivity—reactivity being defined as the change from resting state to task/event. Although it is mentioned as a limitation in the discussion, it is not systematically acknowledged by the authors throughout the manuscript (critical examples being the title, highlights, and conclusion, where only “HRV” is mentioned, and not “resting/trait HRV”). This may be highly misleading for the reader, overgeneralising the findings of the meta-analysis. As acknowledged by the authors in their discussion, vagal reactivity plays a critical role in adaptation and evolution (Beauchaine, 2001; Porges, 2007). This was shown for example in one study cited in the meta-analysis, which originally addressed reactivity and not resting HRV (Laborde and Raab, 2013) and which showed larger effect sizes regarding reactivity in comparison to resting HRV. This is in line with the capability model (Coan et al., 2006) initiated with EEG and applied to HRV/vagal tone by Laborde et al. (2015), where the relationship between physiological variables and cognitive/emotional outcomes proved to be stronger considering reactivity than resting measures. We would then recommend researchers to systematically investigate both resting vagal tone and its reactivity.
3. The third point is linked to the time delay between HRV measurement and self-control performance assessment. According to the theoretical foundations of this meta-analysis, both the neurovisceral integration model (Thayer et al., 2009) and the polyvagal theory (Porges, 2007) would not directly predict an association between resting vagal tone and the outcome of a basic laboratory self-control task realized at a different time point. Even if resting vagal tone is reasonably stable (Bertsch et al., 2012), it can be influenced by many situational factors, such as physical activity (Stanley et al., 2013), meals (Lu et al., 1999), sleeping routine, and time of the day (van Eekelen et al., 2004). These situational factors can in turn influence the performance on self-control tasks. Based on this, even performing vagal tone assessment and self-control performance on the same day but at different time points (e.g., morning and afternoon) might bias the results. Hence, the criterion quality number 9 (i.e., “Assessment of HRV and self-control performance at the same session”) should have not been solely a criterion quality, but an inclusion criterion to the meta-analysis, or at least a moderator. Moreover, it would have been helpful to know how many studies actually met this criterion. The authors mention particularly one study not fitting this criterion, with a time lag of 0 to 61 months between the assessment of HRV and self-control performance. According to theoretical accounts, and the consideration of situational confounding factors, studies not investigating in parallel HRV and self-control should have been excluded from the meta-analysis. We would then recommend researchers to systematically investigate vagal tone together with the self-control outcome. Or in the case both measurements occur at different time points, to provide a sound rationale for this, as well as a detailed report of the situational factors corresponding to when both assessments were realized.

In summary, we highlighted three main issues of this meta-analysis: (1) the need to shift the focus from HRV to vagal tone when investigating self-control, given theoretical accounts; (2) the need to consider systematically vagal tone at rest and its reactivity; (3) the need to having self-control performance assessed together with vagal tone during the same session.

In conclusion, the interpretation of this meta-analysis might require some additional contextual information for the readership to better comprehend its findings. Addressing to some extent this issue, this commentary aimed to serve as guidance for future research endeavors on vagal tone and self-control.

## Author contributions

SL established the first draft of the commentary and EM provided critical insightful comments to improve it.

### Conflict of interest statement

The authors declare that the research was conducted in the absence of any commercial or financial relationships that could be construed as a potential conflict of interest.
